# Key factors behind autofluorescence changes caused by ablation of cardiac tissue

**DOI:** 10.1038/s41598-020-72351-6

**Published:** 2020-09-21

**Authors:** Narine Muselimyan, Huda Asfour, Narine Sarvazyan

**Affiliations:** Department of Pharmacology and Physiology, The George Washington University, 2300 Eye Street NW, Washington, DC 20037 USA

**Keywords:** Biophysics, Biotechnology, Physiology, Cardiology, Optics and photonics

## Abstract

Radiofrequency ablation is a commonly used clinical procedure that destroys arrhythmogenic sources in patients suffering from atrial fibrillation and other types of cardiac arrhythmias. To improve the success of this procedure, new approaches for real-time visualization of ablation sites are being developed. One of these promising methods is hyperspectral imaging, an approach that detects lesions based on changes in the endogenous tissue autofluorescence profile. To facilitate the clinical implementation of this approach, we examined the key variables that can influence ablation-induced spectral changes, including the drop in myocardial NADH levels, the release of lipofuscin-like pigments, and the increase in diffuse reflectance of the cardiac muscle beneath the endocardial layer. Insights from these experiments suggested simpler algorithms that can be used to acquire and post-process the spectral information required to reveal the lesion sites. Our study is relevant to a growing number of multilayered clinical targets to which spectral approaches are being applied.

## Introduction

Percutaneous catheter ablation is currently the mainstream procedure used to terminate atrial fibrillation, as well as many ventricular and nodal rhythm disturbances^1–3^. It is a highly effective and widely accepted clinical treatment that irreversibly ablates the culprit tissue using either cryofreezing, radiofrequency (RF), or laser energy^4^. Yet even with all of the successes that it has achieved, percutaneous catheter ablation has its shortcomings, one being its inability to visualize damaged tissue in real time. The existing means for monitoring local electrical activity, while effective, cannot substitute for direct observation of ablation lesions and the gaps between them. This is because the specific cause of electrical isolation during AF ablation procedures is not clear. It may result from tissue necrosis, diminished excitability of reversibly injured cells, or from temporary edema. When and if edema subsides or reversible injured cells recover, the electrical conduction between the pulmonary veins and the left atrium can be restored. Indeed, despite an initial return to sinus rhythm after RF therapy, AF has a high degree of recurrence^5^. Use of spectral imaging allows one to confirm that the loss of electrical conduction is indeed due to irreversible loss of viable cardiomyocytes. In addition, autofluorescence based imaging is a much better tool to reveal small gaps between the lesions since its spatial resolution is on the order of microns, while electrode-based sensing is limited to a millimeter scale. Standard endoscopic cameras are not well suited for in vivo visualization of ablation-induced damage to the heart muscle due to the presence of the endocardial lining that covers all four cardiac chambers. The main component of the endocardial lining layer is a highly autofluorescent and reflective collagen that obscures spectral changes caused by RF energy to the muscle beneath. The thicker the endocardium, the less observable are the ablation lesions. This is particularly true for the left atrium, where the thickness of the endocardium is the highest among the four chambers of the heart. Moreover, it so happens that the left atrium is where the vast majority of culprits responsible for the most common cardiac arrythmia originate^6,7^.

Our group has recently shown that the use of autofluorescence-based hyperspectral imaging (Auf-HSI) enables the circumvention of limitations imposed by the endocardial collagen layer. This approach employs illumination of the tissue surface with UV light while acquiring grayscale images across multiple wavelengths within the visible range. This creates a three-dimensional dataset, where *x* and *y* are the two spatial dimensions, while wavelength λ stands for the third dimension. Autofluorescence spectra are extracted from each x_i_,y_i_ pixel. Then, various mathematical algorithms are used to sort the pixels based on subtle differences in their normalized spectral profiles. Thereafter, the individual pixels are assigned custom pseudocolors to distinguish the ablated from the uninjured tissue^8^.

To date, we have confirmed that Auf-HSI can reveal ablation lesions made in the left atrium of large mammals, including pigs, sheep, and cows^9^, as well as in donated human heart tissue^10^. The dimensions and the shape of the lesions delineated by Auf-HSI were in perfect agreement with the conventional post-ablation staining methods such as TTC^10–14^. These promising bench findings have led to our ongoing efforts to incorporate Auf-HSI technology into a percutaneous imaging catheter^11,15,16^. The design of this catheter includes a saline-filled balloon to displace optically dense blood from the endocardial surface. It also includes insertable fiber optic bundles to deliver UV light and to collect the emitted visible spectra. An alternative approach is to integrate multiple single-point optical sensors directly into the tip of existing ablation catheters^17^. In the latter case, the autofluorescence profiles from individual points can be obtained by simply touching the tissue without the need for an inflatable balloon to create an optical window.

This paper examined the key physical factors behind spectral changes in cardiac tissue autofluorescence profiles caused by RF ablation. Equipped with such knowledge, we then identified specific wavelength ranges where ablation-induced changes are the most pronounced and consistent, allowing us to simplify both acquisition and post-processing algorithms. Insights from our studies can be applied not only to the heart, but also to other multilayered body tissues where spectral imaging offers diagnostic promise, including the skin, endovascular or epithelial surfaces.

## Materials and methods

### Tissue sources and ablation procedures

To fully cover the range of endocardial layer thickness reported in human subjects^18,19^, we performed experiments using freshly excised hearts from three different species. These included rat ventricles, where the endocardial thickness is negligible, and ending with market-age cows, where the endocardial layer in the left atrium can reach one millimeter in thickness. To test the effects of storage conditions on NADH and other muscle fluorophores, we used fresh rat ventricles, which enabled data acquisition immediately after the excision of the tissue. Bovine and porcine hearts were obtained from a local abattoir or after surgical training at the Washington Institute of Surgical Education and Research. The choice of the species was based on our published study in which we compared atrial tissues from market age animals to human samples^9^. Different aspects of human atrial anatomy had similarities to either porcine or bovine samples. For example, wall thickness was similar to values from market-aged pigs, while endocardial layer thickness was similar to that of a cow. The explanted hearts were transported to the laboratory on ice within a 2–3 h window after the excision, followed by dissection to expose the relevant surfaces to be ablated. RF energy was delivered with a non-irrigated ablation catheter (EP Technologies, Boston Scientific, Marlborough, MA, USA). The 4 mm ablation tip was placed perpendicular to the endocardial surface, with ablation durations varying from 5 to 30 s and tip temperatures ranging between 50 and 70 °C. These settings created lesions similar in size to those placed during clinical RF ablation therapy, as detailed previously^8^.

All animal protocols were approved by the George Washington University Institutional Animal Care and Use Committee. Experiments were performed in accordance with the United States Association for Assessment and Accreditation of Laboratory Animal Care guidelines and regulations.

### Imaging hardware

The LED source (Precision LED Spotlight from Mightex, Pleasanton, CA—either 365 nm UV or 5500 K cool white) was placed ~ 5 cm away from the tissue surface and positioned to reduce specular reflection. Hyperspectral datasets were acquired using a commercial HSI system (Nuance FX, PerkinElmer/Cri, Waltham, MA, USA) fitted with Nikon AF Micro-Nikkor 60 mm f/2.8D lens. The Nuance FX system comprises a liquid crystal tunable filter (CRi LCTF) and a monochromatic charged coupled device (Sony ICX285 CCD). The Nuance FX system can capture wavelengths between 420 and 720 nm with a spatial resolution of 1,392 × 1,040 pixels. For the trans-illumination experiments, a large piece of bovine endocardium was dissected from the underlying muscle tissue and placed on top of a flat-surfaced LED lightbox. Transmission images were acquired at 700 nm using the Nuance FX optical density mode settings. The thickness values were derived from the optical density numbers and calibrated using direct caliper measurements, as described earlier^9^.

### Post-processing of hyperspectral imaging datasets

The Nuance FX software package was used to perform supervised linear unmixing, with green and red pseudocolors assigned to ablated and unablated tissues, respectively. A typical signal processing protocol involved the extraction of spectra from regions of interest from the unablated and ablated tissues, followed by spectra normalization from 0 to 1 and then finding the difference between them. This difference was then corrected for the spectral sensitivity of the CCD and CRi LCTF of the Nuance FX system to facilitate compatibility of our conclusions to other acquisition systems. A detailed description of these calculations can be found in our earlier publication^8^. For brevity, the acronym DBNS was used throughout the article in lieu of ‘Difference Between Normalized Spectra’.

### Statistical analysis

Student’s t test was used to evaluate statistical significance. Values are presented as mean ± standard error of the mean, with p < 0.05 considered as statistically significant. The Pearson’s correlation coefficient was used to determine the statistical significance of the relationships between the endocardial layer thickness and the amplitude of the DBNS at individual wavelengths.

## Results

### Major fluorophores of cardiac tissue before and after RF ablation

**Figure 1:**
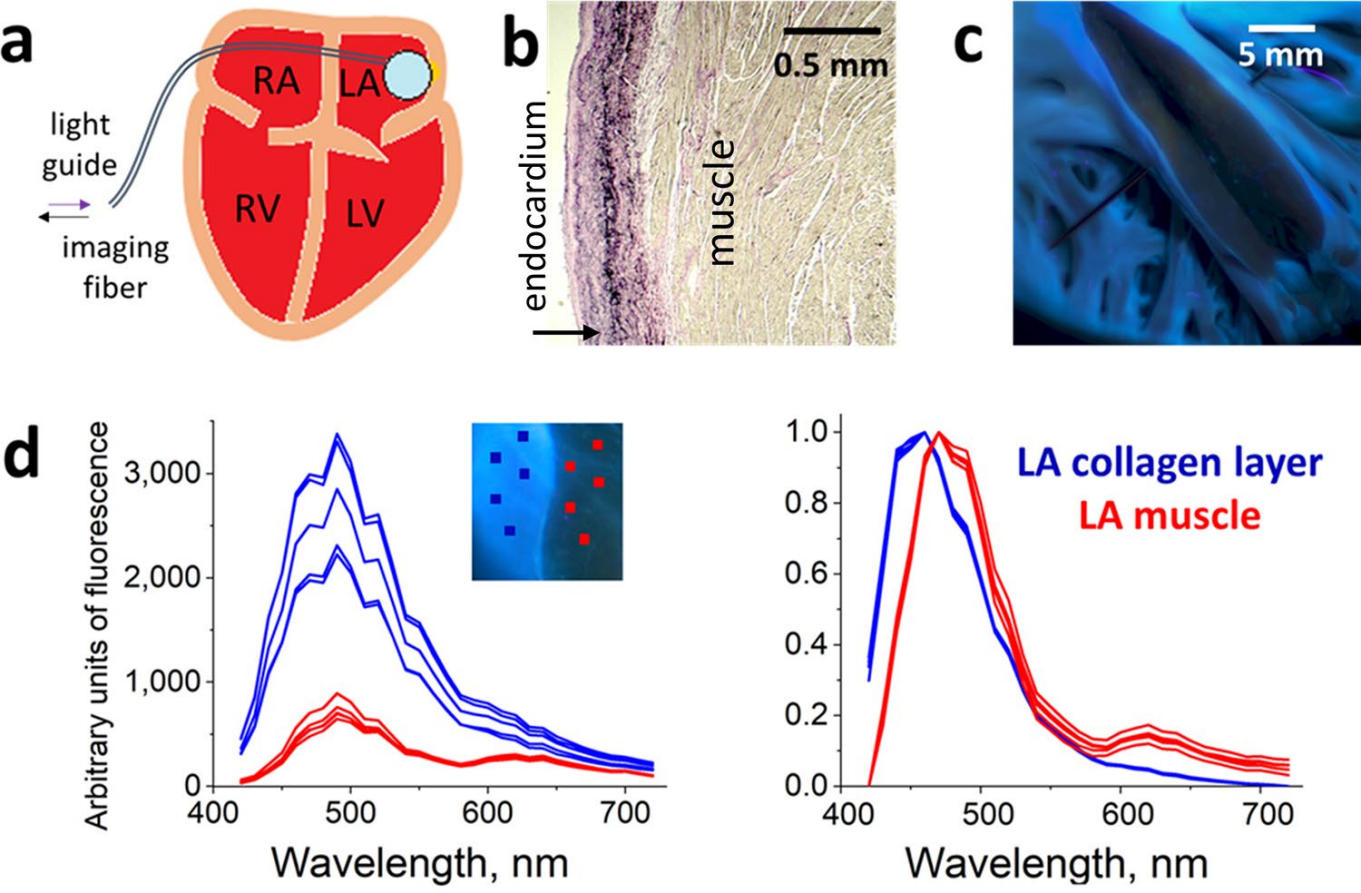
The two main layers facing the ablation catheter and their respective spectra. (**a**) Depiction of a percutaneous imaging catheter and its positioning against the endocardial left atrial (LA) surface. (**b**) Histology of the LA wall showing the endocardial collagen layer and the muscle beneath it. (**c**) A piece of porcine LA under UV illumination. A cut was made in the middle to expose the muscle layer to show visually how much dimmer the muscle autofluorescence is when compared to the endocardial collagen layer. (**d**) Raw spectra of endocardial collagen layer and the muscle beneath it from porcine LA (on the left). Individual traces are extracted spectra from regions of interests (ROI) shown in the insert. On the right side are the same traces after normalization and adjustment for Nuance FX sensitivity. These two sets of graphs illustrate how dramatically spectral normalization reduces the variability of profiles from individual ROIs.

Cardiac muscle is covered by epicardial and endocardial layers of connective tissue, made predominantly from interwoven collagen fibers. In this study, we present data mainly from the endocardial surface of the left atrium (LA), where most ablations take place clinically and where the endocardium is the thickest. Spectral responses from ablation sites in other cardiac locations, including ventricular sites or epicardial surfaces, were conceptually similar (data not shown). Figure 1a,b illustrate the general structure of the cardiac wall to be ablated. Two structural components of the heart surfaces are facing the ablation catheter: a layer of connective tissue and a layer of cardiac muscle beneath it. When sufficient RF energy is applied, it penetrates all the way to the underlying muscle with the goal of destroying the cells that act as arrhythmogenic culprits. The major endogenous fluorophore inside the muscle cells is mitochondrial NADH with excitation/emission peaks of around 350 and 460 nm, respectively^20,21^. Once the integrity of the cardiac muscle cells is destroyed due to RF or other means of ablation, the NADH levels irreversibly decline^13^. The secondary fluorophores found in cardiac muscle cells include flavins, flavoproteins, lipids, lipofuscin, and lipid–protein conjugates^18,22^. They fluoresce at longer wavelengths (< 500 nm), but their overall signal from the unablated muscle is weaker compared to the NADH signal. Hereafter, we will refer to them simply as ‘yellow pigments’.

When looking at the muscle spectra shown in Fig. 1d, one can also notice an apparent ‘peak’ around 630 nm. This ‘peak’ is caused by an increased photon absorption by heme-containing myoglobin. Muscles appear red under white light illumination due to an abundance of myoglobin which absorbs ‘green’ and ‘yellow’ photons from 520 to 600 nm range^23^. The presence of myoglobin also creates a dip in the same ‘green-yellow’ range of the spectrum emitted by NADH and other cardiac UV excitable fluorophores, leading to the apparent 630 nm ‘peak’.

The main structural component of the endocardial layer is collagen. The latter is a highly fluorescent molecule with a broad absorption peak of around 300 nm^24^. Even when illuminated with 350–370 nm UVA light (which is closer to the absorption peak of NADH), the intensity of the endocardial layer autofluorescence is still much higher than that of the muscle layer beneath (Fig. 1c). At this excitation range, the normalized emission spectra of these two layers largely overlap (Fig. 1d). When the endocardial collagen layer is heated during the RF ablation process, the amplitude of its autofluorescence transiently declines, only to quickly return to the original levels once the tissue cools down. As a result, when the LA endocardial surface is imaged post-ablation, the changes in its autofluorescence spectrum are minimal.

Paradoxically though, while the presence of the endocardial collagen layer obscures the drop in muscle NADH, it does, on the other hand, help to reveal an increase in the light scattering by the ablated muscle below. These two factors affect the difference between the normalized spectral profiles of native and ablated LA tissue. To understand the mechanism better, let us first examine how such difference traces were derived.

### Differences between normalized spectra of native versus ablated cardiac tissues

**Figure 2:**
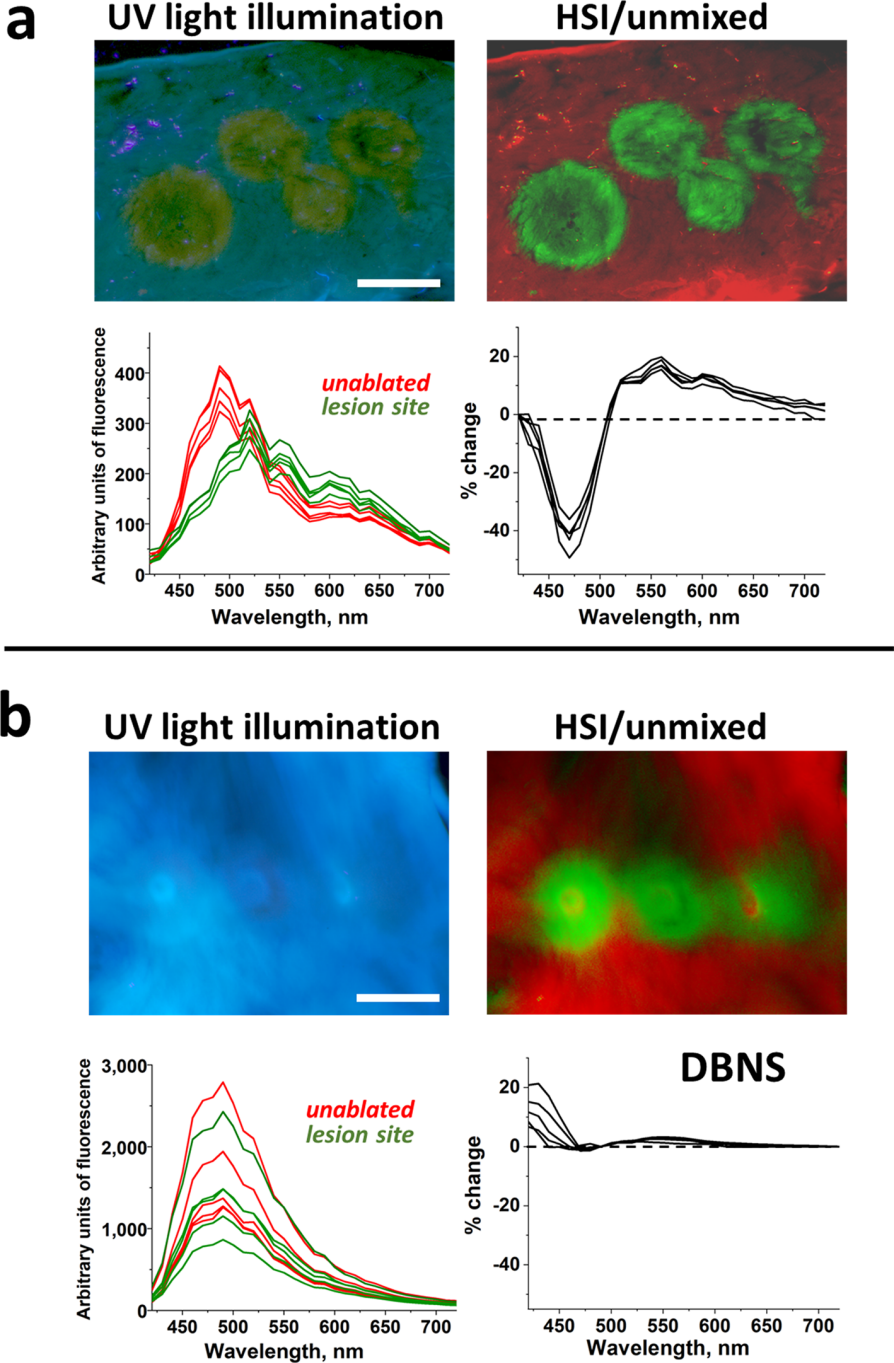
Spectral profiles extracted from RF-ablated ventricular muscle and LA surface. (**a**) Top left: Visual appearance of ablated bovine ventricular slab with several lesions. Scale bar 5 mm. Top right: corresponding composite image from spectral unmixing of the Auf-HSI dataset, with the green and red pseudocolors representing native and ablated tissues, respectively. Bottom left: raw traces from lesions and neighboring tissue before any processing. Bottom right: difference traces after raw traces were normalized and adjusted for equipment sensitivity. (**b**) The same as above but for ablated bovine LA with a thick collagen layer. Scale bar 5 mm.

In the absence of a collagen layer, the differences in the autofluorescent profiles of native and ablated tissues illuminated with UV light are very pronounced. To illustrate this, Fig. 2a shows the visual appearance of a cross-sectioned bovine ventricular muscle illuminated with UV light. RF ablation of muscle leads to a prominent drop in the 450–480 nm range of the autofluorescence spectrum, which makes lesions appear yellowish on a blue background. The graph on the bottom left shows the raw spectral profiles taken from eight regions of interest (ROIs): four from the lesion sites and four from the proximal unablated tissue. The black traces on the right show the differences between normalized spectra from the ablated and the adjacent unablated sites. We refer to these traces as DBNS. They are obtained by normalizing the raw spectra and adjusting them for the spectral sensitivity of the Nuance FX hardware. The greater the amplitudes of the DBNS and the more similar they are to each other, the better the outcomes of the post-acquisition unmixing. When ablations are done on ventricular muscle, the DBNS are very pronounced and uniform (Fig. 2a). The use of hyperspectral imaging enhances the visual appearance of the lesion sites, although they can also be observed by an unaided eye (compare Fig. 2a top left vs. top right images).

The situation changes considerably when RF lesions are made on cardiac muscle covered by a collagen layer thicker than ~ 100 μm. This is true for large mammals, including humans, where collagen layer thickness varies across the LA endocardial surface, ranging from 0.1 to 0.8 mm^9^. Figure 2b shows an extreme case of DBNS from lesions made on bovine LA, where collagen layer thickness approaches 1 mm. The amplitudes of the raw autofluorescence profiles from the individual pixels become much more variable (Fig. 2b bottom left), while the DBNS are less pronounced (Fig. 2b bottom right). The drop in the 450–480 nm range disappears entirely and there is a noticeable rise within the 420–450 nm emission range. Although the amplitudes of these DBNS are much smaller and less consistent compared to the DBNS shown in Fig. 2a, post-acquisition processing of this hyperspectral dataset still enables distinguishing of the lesions (Fig. 2b top right). This was possible because the initial raw traces were acquired from the lesions made on the *same* piece of tissue. Therefore, all other conditions that can affect spectral difference between ablated and unablated tissues (and, consequently, the shape of DBNS), such as dissimilarities in tissue composition from different animals, time since heart excision, and storage conditions, were identical.

**Figure 3:**
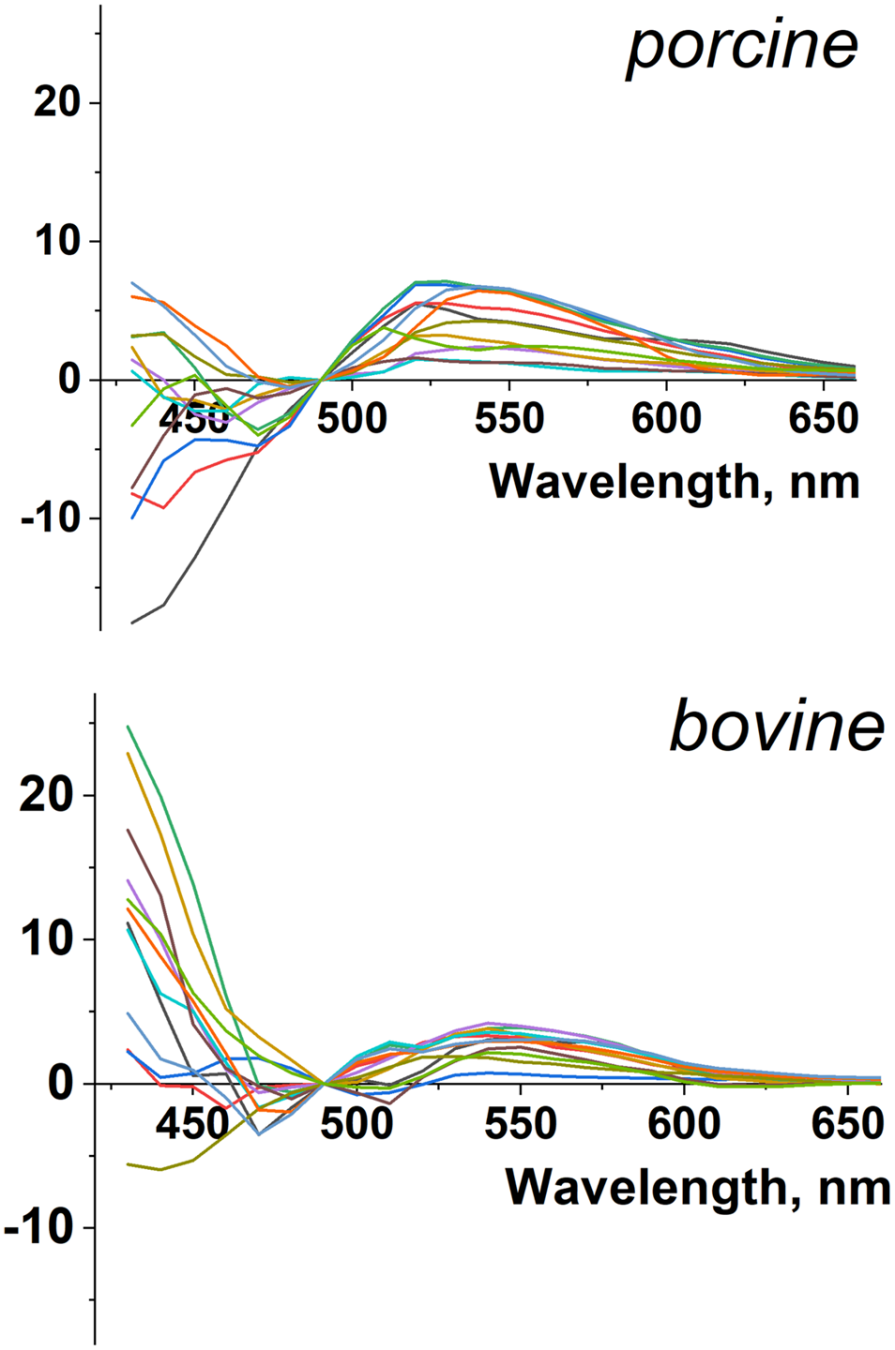
Variability of DBNS traces. Twelve traces showing variability of DBNS from different lesion sites and/or individual preparations from species with endocardial layer thickness comparable to human subjects.

However, much larger DBNS variability is expected between the samples from different individuals. This is because neither the subject nor the imaging conditions will be identical. Figure 3 illustrates such variability by showing the DBNS of different lesions from four porcine and bovine LAs. Such variability in DBNS traces interferes with the ability to use pre-acquired spectral libraries or other processing algorithms to reliably classify the pixels. Therefore, it is important to understand the main factors that can contribute to DBNS variability. By taking these factors into account, better protocols to acquire Auf-HSI datasets can be designed, whether for bench experiments or in vivo ablation procedures.

### Main factors that impact changes in the autofluorescence spectrum of ablated cardiac surface

**Figure 4:**
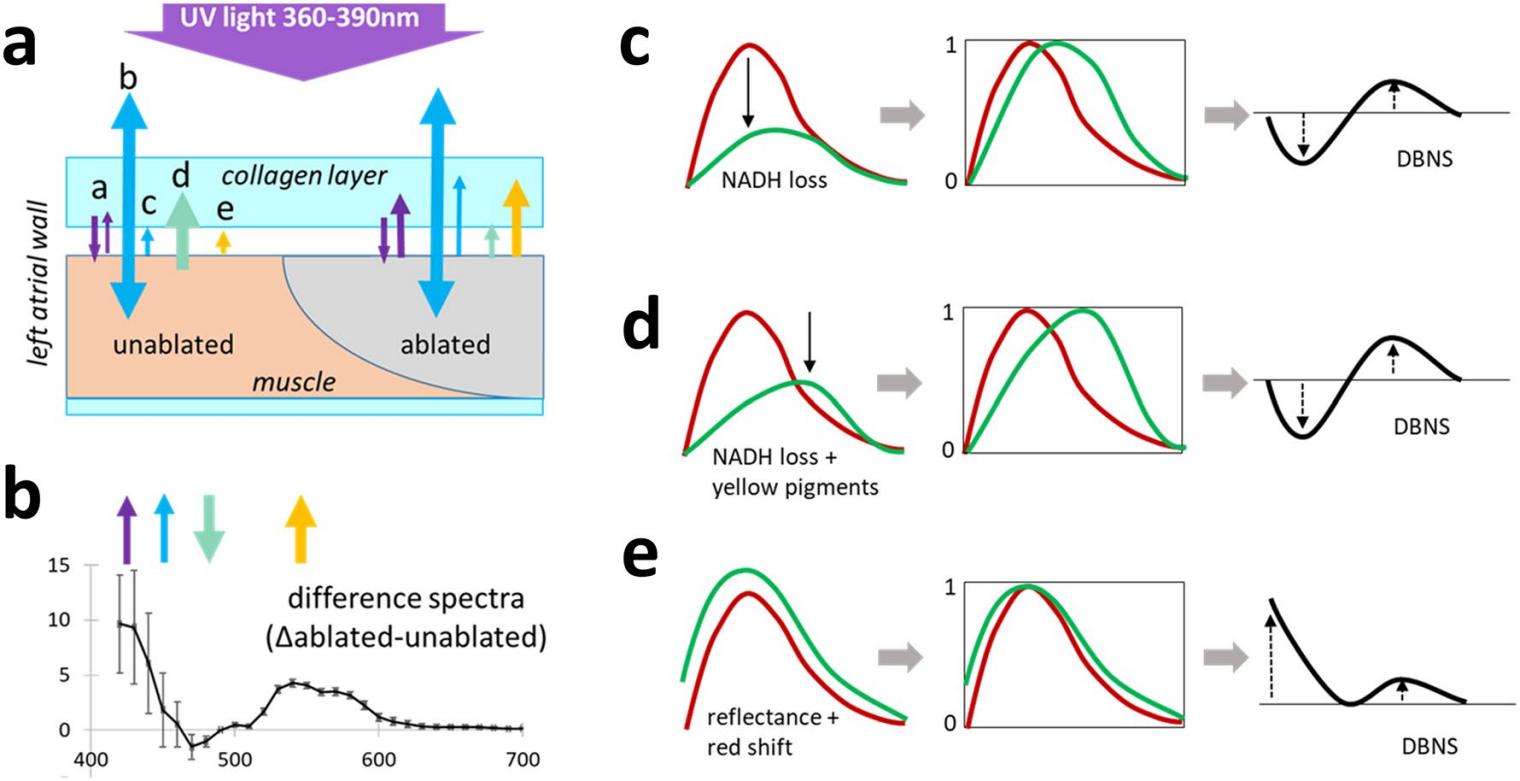
Main contributors to the spectra of ablated and unablated atrial surface. (**a**) Cartoon depicting cross-section of an atrial wall. The arrows point to: a) fraction of illuminating light reaching the muscle and reflected back to the detector; b) collagen autofluorescence (440–480 nm); c) collagen autofluorescence reflected by the muscle layer; d) NADH fluorescence from live muscle (460–490 nm); e) yellow pigments (550–600 nm). (**b**) Average DBNS from several lesions made on porcine LA showing where the above factors impact the difference spectra. (**c**) C–E. Simplified cartoons that illustrate the effects of individual factors on autofluorescence profiles of native (red trace) and ablated (green trace) tissue before and after normalization. The resulting DBNS traces are shown on the right. The dotted arrows point to the overall direction of changes.

Figure 4A depicts our current understanding of the interplay between multiple variables impacting the overall spectral response to the illumination of cardiac surfaces with UV light. The thickness of the upper collagen layer determines the intensity of its autofluorescence. The latter acts as a secondary light source that illuminates the muscle layer below. The optical response of the muscle layer depends on the degree of its thermal damage. Specifically, lesion severity determines: (1) the amplitude of changes in the muscle autofluorescence caused by the loss of NADH and the rise in yellow pigments and (2) the degree with which the surface of ablated muscle reflects back photons coming from the endocardial collagen layer on top of it. The latter is a product of the layer thickness and collagen autofluorescence intensity. Any reflected taillight from the UV source might also be at play. The total amount of light coming back to the detector from the muscle layer is then affected by the thickness of the endocardial layer through which these photons must travel. In the next sections, we will present the results of experiments that examined how individual factors, denoted by the colored arrows in Fig. 4a,b, can affect the shape of DBNS. In these sections, we will be referring back to the Fig. 4c–e cartoons that depict the overall directions of changes that these individual factors impose on DBNS traces.

### Ablation-induced drop in NADH autofluorescence

**Figure 5:**
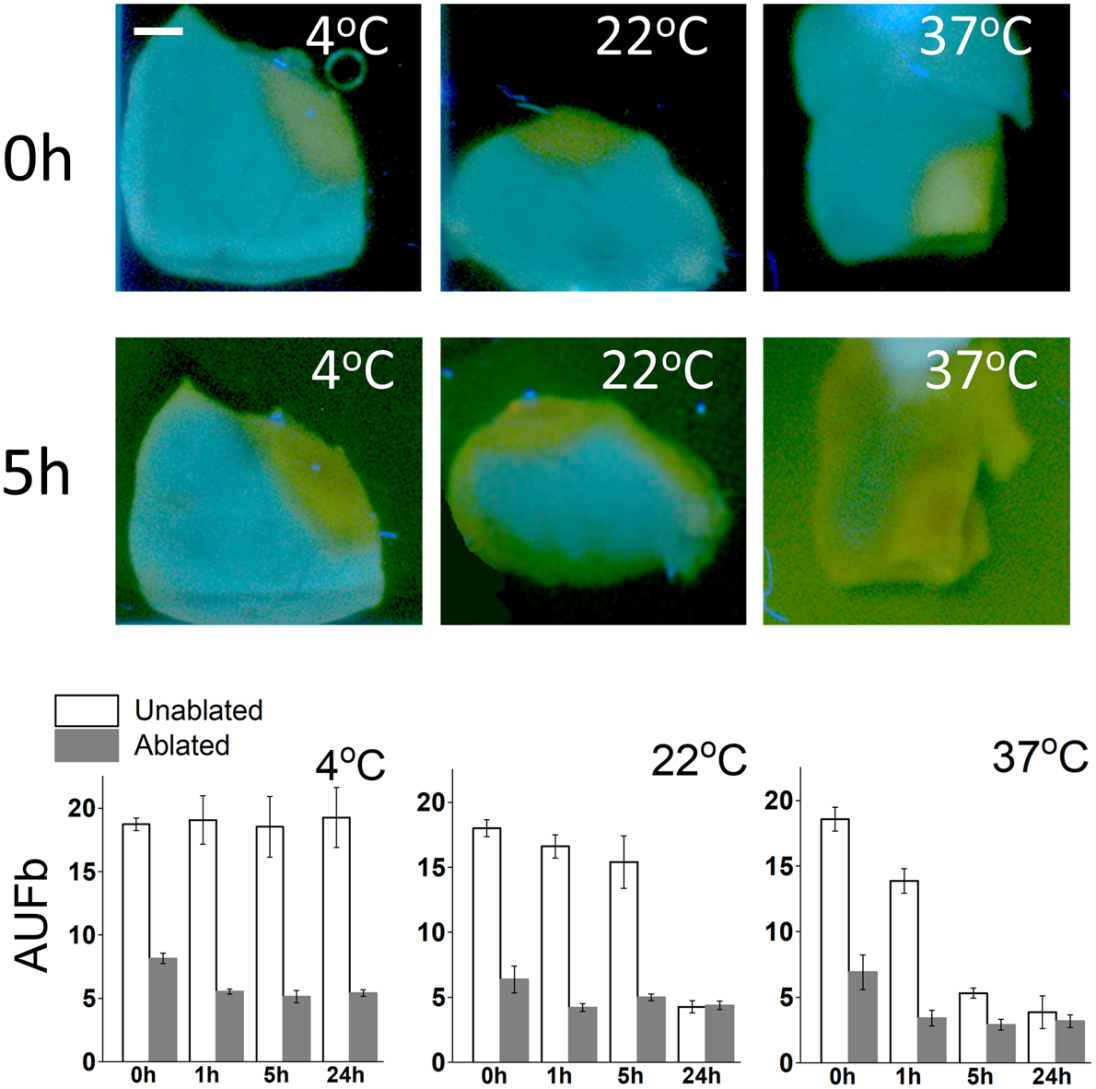
Decline of cardiac muscle NADH during storage. Top row: visual appearance of an ablated rat ventricle cut into several pieces and stored at different temperatures under UV illumination. Scale bar 2 mm. Representative images from three separate experiments are shown. Bottom row: the graphs depict the intensity values at 460 nm extracted from Auf-HSI cubes.

As reported by many, including our group, ischemia leads to increased levels of NADH fluorescence in both blood and saline-perfused heart preparations^25–27^. This is because only the reduced form of the molecule exhibits significant autofluorescence and the latter accumulates when oxygen is not available to pass electrons down the mitochondrial redox chain. After heart tissue is excised from an animal and/or taken off the perfusion system, its muscle cells gradually die, leading to a steady decline in tissue NADH levels. The rate of such decline depends on both the duration of storage and on temperature. To illustrate this effect, we ablated freshly excised saline-perfused rat ventricles, cut them into semi-equal pieces and kept them at 4, 22, and 37 °C while acquiring spectra at multiple time points. The levels of NADH autofluorescence remained stable for hours when the samples were kept at room temperature (Fig. 5) and for several days when the samples were kept on ice. By contrast, background autofluorescence rapidly declined in samples stored at 37 °C and, with it, the difference between the ablated and unablated tissues. Therefore, if tissue samples are not stored or transferred to the lab at the proper temperature, the NADH levels would start out low at the beginning of the experiment. Consequently, a much smaller drop in the 450–480 nm range will be recorded during the on-the-bench ablation experiment. On the other hand, if one performs ablation on a piece of freshly excised heart that is in the process of becoming ischemic, the ablation-induced NADH drop can be exaggerated.

Therefore, the exact amplitude of the NADH drop is affected by multiple factors including: (1) amount of applied RF energy, (2) degree of tissue ischemia, and (3) storage conditions, including duration and temperature. However, regardless of its exact amplitude, NADH loss will always impact DBNS traces in the way shown in Fig. 4c.

### Ablation-induced release of flavins and lipid peroxidation products

**Figure 6:**
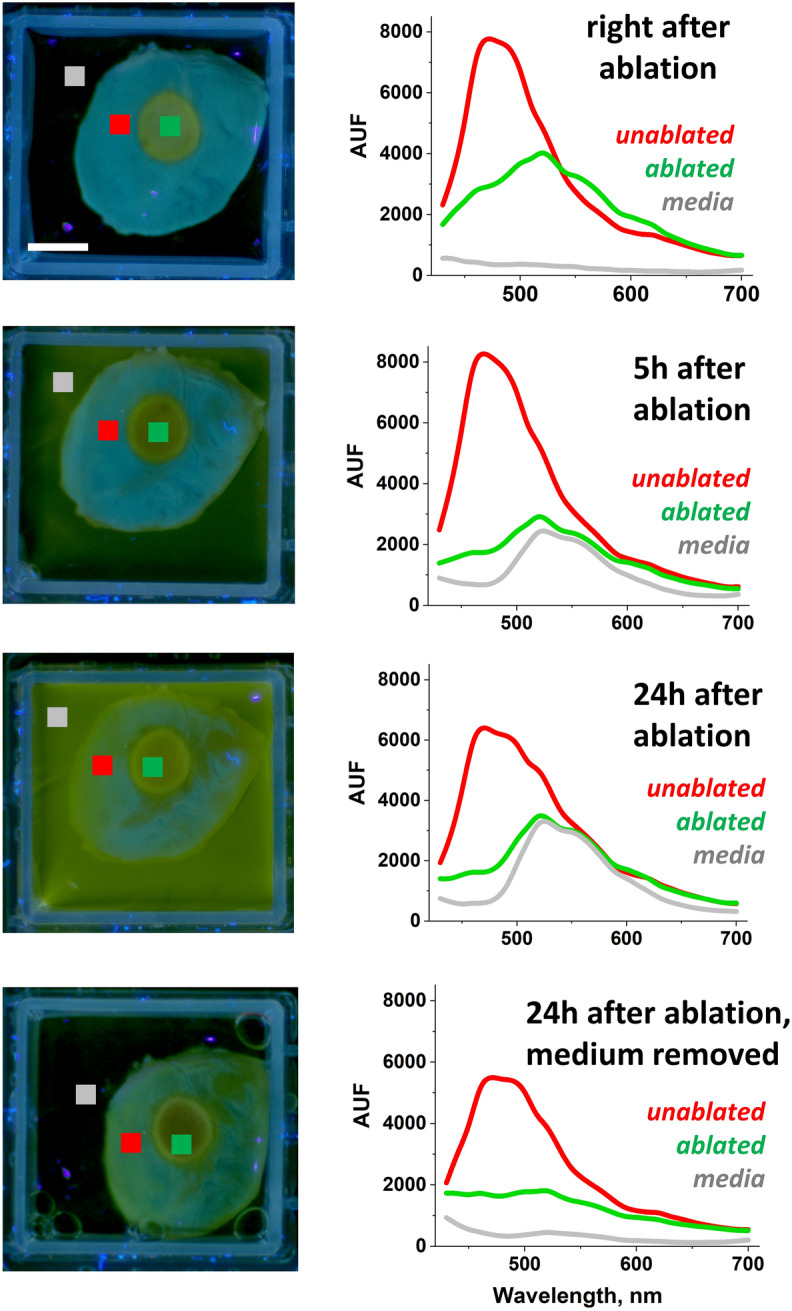
Formation and release of yellow pigments. Immediately after excision from an anesthetized rat, the heart was perfused with saline and RF ablated. Auf-HSI datasets were taken at different time points while the hearts were kept in saline at room temperature. The traces next to the RGB images are extracted Auf-HSI spectra from shown ROIs placed on native and ablated muscles and the surrounding medium. They show a gradual decline in tissue NADH levels and the release of yellow pigments into the medium. Scale bar 5 mm. Representative images and traces from three separate experiments are shown.

RF energy heats tissue, which enhances the formation and release of secondary fluorophores from cardiac muscle cells. To demonstrate this process directly, we performed an experiment shown in Fig. 6. RF lesions were made on the left ventricles of freshly excised saline-perfused rat hearts. The hearts were immersed in saline and Auf-HSI datasets were acquired immediately at 5 and 24 h after ablation. At the end of 24 h, the media was replaced by fresh saline and the samples were re-imaged. The autofluorescence spectra were then extracted from the pixels corresponding to the unablated (red trace) and ablated (green trace) surfaces, as well as from the media (gray trace). The representative images and spectra next to them show an immediate ablation-induced drop in NADH, followed by a slow release of heat-induced yellow pigments into the media. At the end of 24 h, the spectrum of the media becomes identical to the spectra of the ablated muscle. Multiple compounds are believed to be responsible for the heat-induced appearance of yellow autofluorescent products, including flavins^28,29^, lipofuscins^30^, lipid–protein additives, and peroxidation products^31,32^. Their formation contributes to the DBNS elevation in the 520–600 nm range, an effect observed in all tested species. The direction of wavelength-specific changes in the DBNS traces caused by the heat-induced release of yellow pigments is shown in Fig. 4d.

### Reflection of endocardial collagen autofluorescence by the underlying muscle layer

**Figure 7:**
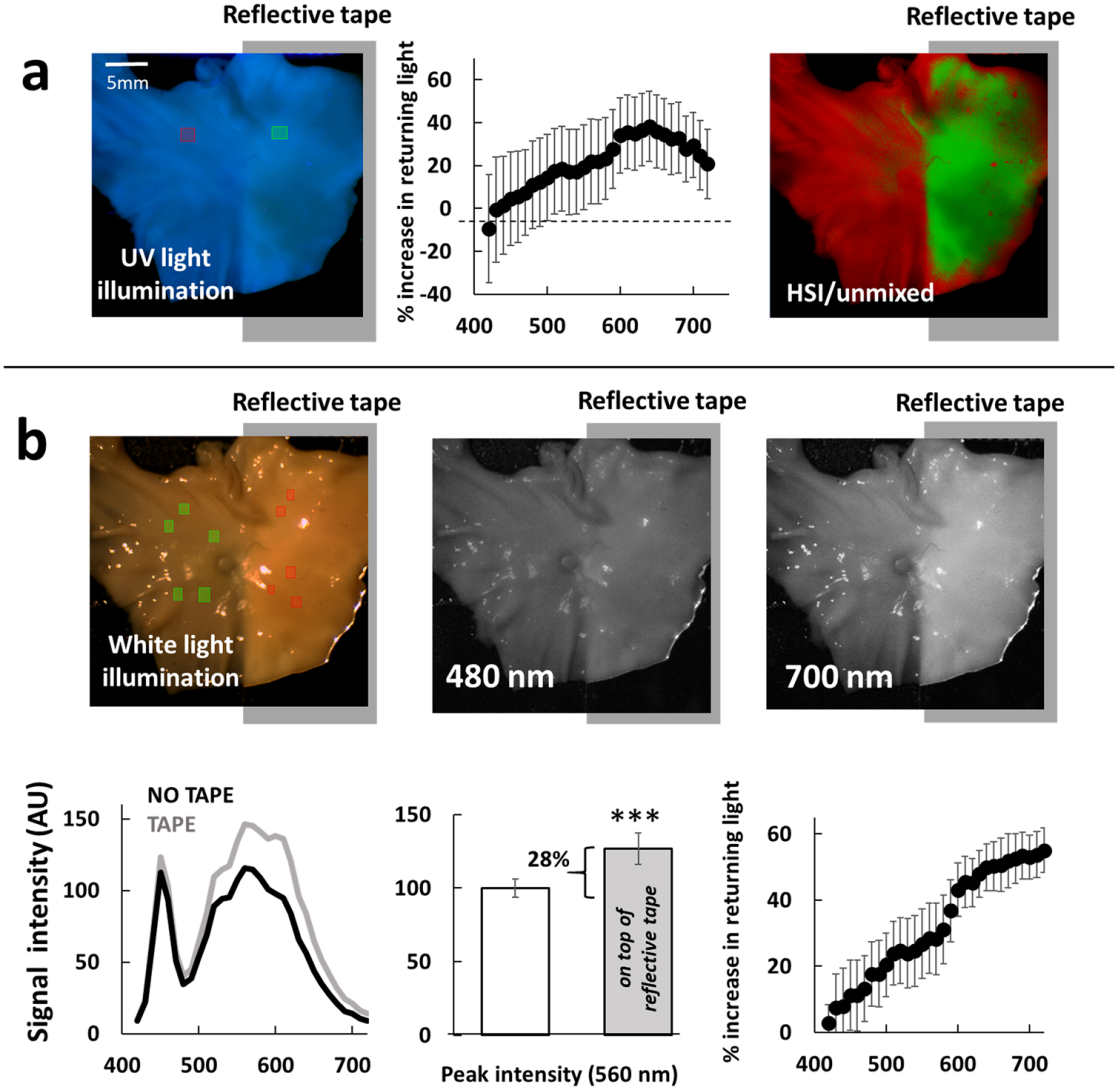
Effect of the reflective surface beneath the endocardial layer. (**a**) Appearance of a dissected bovine endocardium placed on top of a reflective tape and illuminated with 365 nm UV LED. Left: visual appearance of the piece under UV light. The ROIs on each side were used to unmix the Auf-HSI dataset (center). Right: percent of intensity increase at each wavelength calculated as (F_tape_ − F_no tape_)/F_no tape_ × 100. (**b**) Upper row: appearance of detached bovine endocardium placed on top of reflective tape and illuminated with a broadband white LED light. The images taken at 480 and 700 nm help to visually illustrate the increased amount of returning photons at longer wavelengths. Bottom row: left: raw spectra showing the increased amplitude of the signals from the right side. The shape of the spectrum corresponds to the Mightex 5500 K profile. Center: percent of increase in peak intensity at 560 nm averaged from 5 different ROIs. Right: relative increase in % of photons at each wavelength calculated as (F_tape_ − F_no tape_)/F_no tape_ × 100.

As we and others have previously reported^14,33^, RF ablation increases the diffuse reflectance of the muscle due to an increase in its scattering coefficient, primarily due to protein coagulation. To directly illustrate the effect of increased reflectance by the bottom layer on DBNS traces, we placed a piece of dissected bovine endocardium on top of a reflective tape, followed by Auf-HSI. The reflective tape beneath the endocardial layer led to a small increase in overall signal amplitude and a small, yet consistent red shift in the normalized spectra of the returning spectra, which enabled the successful classification of the pixels on top of the reflective tape (Fig. 7a). To make these two effects more visually evident to the reader, we conducted a similar experiment using a broadband white LED light (Mightex 5500K) instead of UV LED. In the former case, it was not the autofluorescence of endocardial collagen layer, but photons from white LED that penetrated the layer and got reflected back. Figure 7b shows an increase in the overall amplitude of the reflected light on the side of the reflective tape, with an elevated amount of returning photons at longer wavelengths visually evident (compare images from the 480 nm vs. 700 nm spectral bands extracted from a hyperspectral dataset).

We argue that something similar, albeit smaller in amplitude, occurs when a layer of collagen is present on top of the ablated muscle. Specifically, the ablation-induced increase in muscle diffuse reflectance causes: (1) increased contribution of collagen layer autofluorescence to the amplitude of the overall spectrum and (2) a red shift in the normalized spectral profiles. The latter is due to an increased proportion of photons with longer wavelengths that can pass through the collagen layer on their way back to the detector. The overall direction of wavelength-specific changes in DBNS traces caused by the increased reflection of endocardial collagen autofluorescence by the ablated muscle below is shown in Fig. 4e.

### Impact of endocardial collagen layer thickness

**Figure 8:**
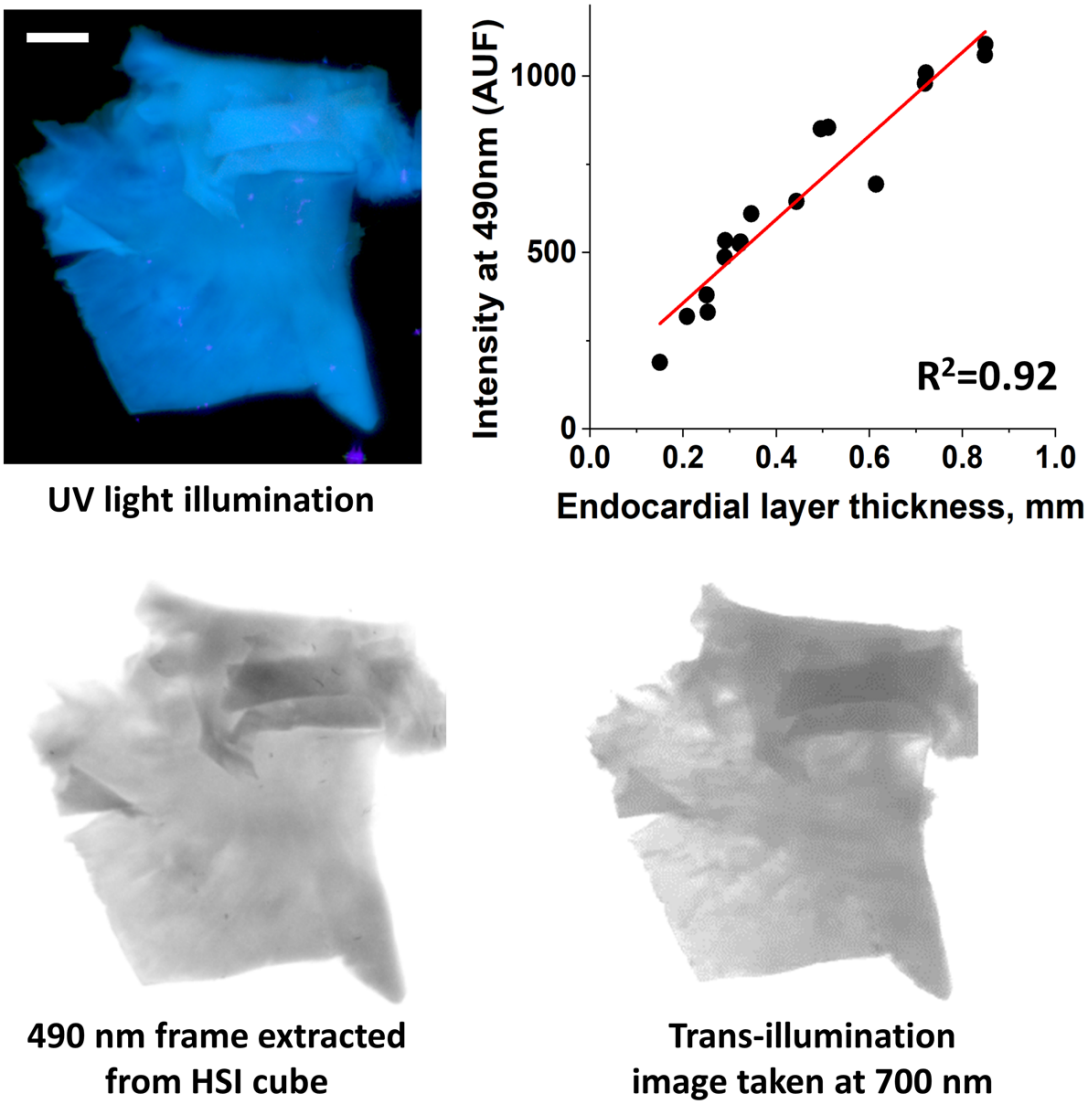
Correlation between collagen layer thickness and its autofluorescence. A piece of detached endothelium was folded several times to double and triple its thickness. Caliper measurements were used to translate OD values to microns. The graph on the top right shows a near linear correlation between collagen layer thickness and its autofluorescence. Collagen autofluorescence was derived from intensity values of the 490 nm frame of the HSI cube. Thickness values were estimated using transmission light measurements at 700 nm. Scale bar 5 mm.

To quantify how the thickness of the endocardial collagen layer impacts the amplitude of the acquired spectra and, consequently, the shape of the DBNS traces, we conducted two sets of measurements. First, we quantified the relationship between the thickness of the endocardial layer and the intensity of its autofluorescence. The endocardium was carefully dissected from the underlying muscle and folded in several places. This was followed by thickness assessment and concurrent Auf-HSI. The thickness values at each pixel were obtained from the optical density measurements using a trans-illumination setup. Maximum autofluorescence intensity was then derived from the Auf-HSI dataset. The data shown in Fig. 8 confirmed that autofluorescence intensity is nearly linearly proportional to the endocardial layer thickness—up to almost 1 mm. Considering that the amplitude of *muscle* autofluorescence is 4–5 times lower than that of endocardial collagen (see Fig. 1d, middle), these measurements implied that the absolute values of the peak autofluorescence across the LA surface can serve as a first-order quantitative indicator of endocardial layer thickness.

**Figure 9:**
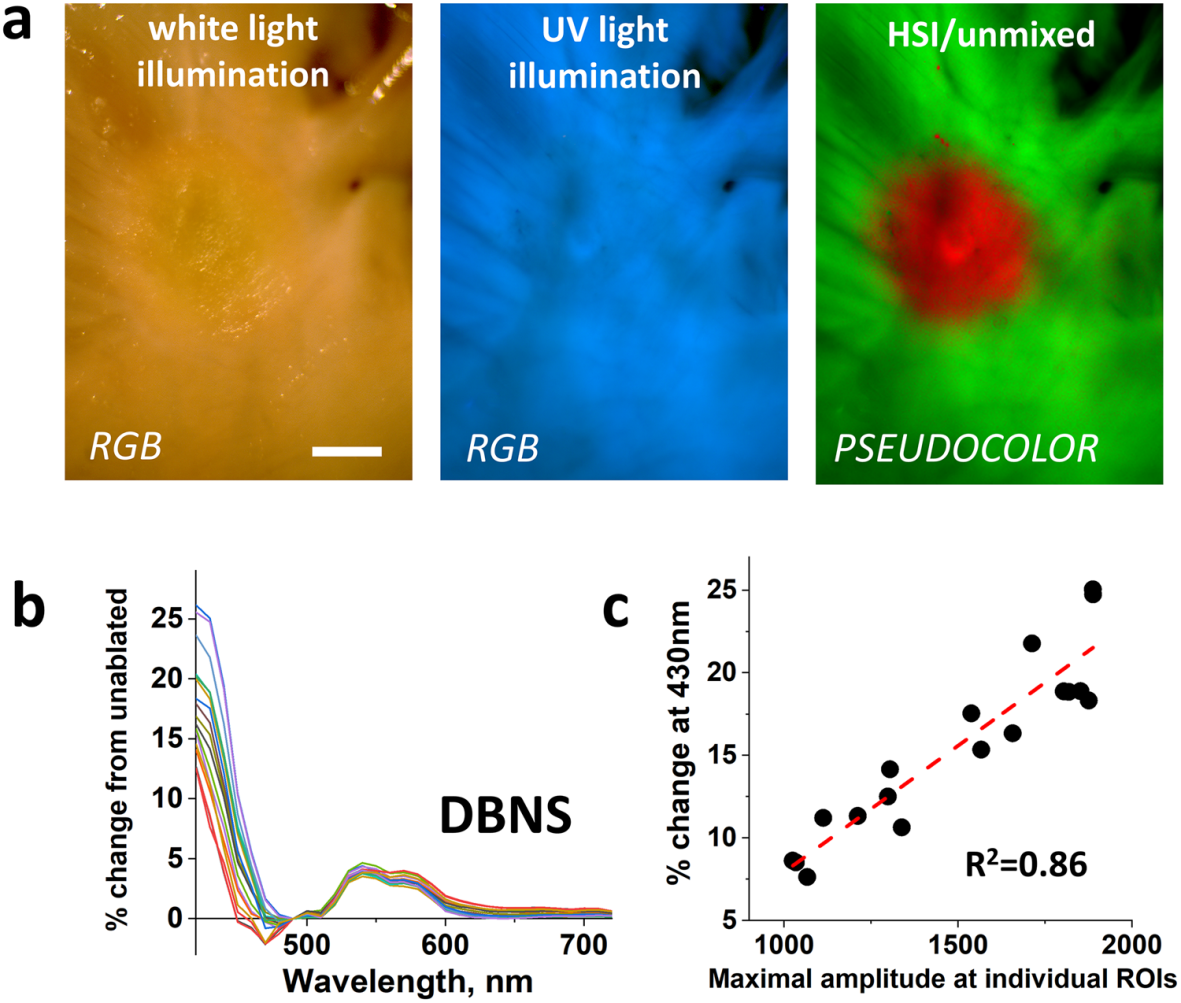
Impact of collagen layer thickness on DBNS spectral shifts. (**a**) A closeup of bovine LA with a RF lesion under white light and UV illumination. Scale bar 2 mm. On the right is the ablation lesion revealed by Auf-HSI approach. (**b**) Spectra from eighteen 5 × 5 pixel ROIs acquired to plot multiple DBNS. (**c**) The graph shows a linear correlation between the values of DBNS shift at 430 nm and the intensity values at 490 nm. The latter serves as a first order estimate of collagen layer thickness.

The second experiment involved a detailed analysis of an ablation lesion made on LA with a particularly uneven endocardium averaging about 200 μm in thickness (Fig. 9a). Spectra from multiple ROIs within the lesion were acquired (Fig. 9b). Values of DBNS at each wavelength were then plotted against the peak amplitude of the spectrum. As mentioned in the previous paragraph, the latter can serve as an indicator of endocardial thickness at each specific ROI. This analysis revealed a highly significant positive correlation between DBNS shift for wavelengths in the 420–480 nm range and the edocardial layer thickness (Fig. 9c). These data indicate that the thicker the endocardium, the higher is the elevation of the left shoulder of DBNS.

### Optimization of acquisition and post-processing algorithms based on DBNS traces

**Figure 10:**
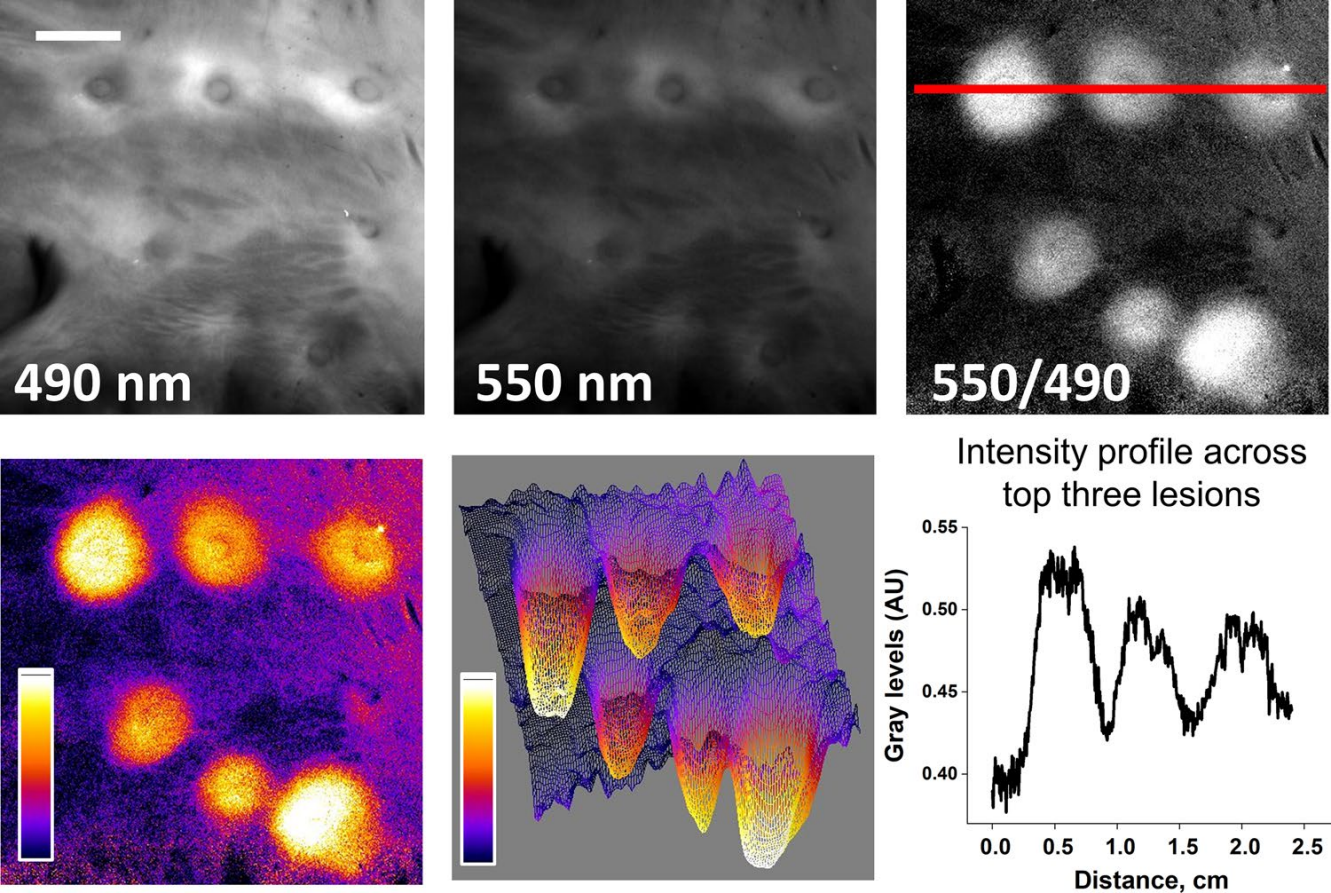
Dual-wavelength approach revealing LA ablation lesions made on highly collagenous LA tissue. The top row shows images from 490 and 550 nm spectral bands extracted from the Auf-HSI dataset and the ratio between them. The bottom row shows the same ratio image in pseudocolor and as a 3D mesh. The graph illustrates the intensity profile across the red line from the ratio image above. Scale bar 5 mm.

A closer examination of the ‘up’ and ‘down’ trends across the DBNS traces (Fig. 4c–e) reveals that the three main variables have opposing effects on the 420–480 nm range. At the same time, all three variables, including drop in NADH, release of yellow pigments, and red shift in the reflected endocardial collagen spectra, lead to an elevation in the 500–600 nm range. This suggests that spectral changes in the 500–600 nm range can serve as a more reliable index to reveal ablated cardiac muscle in samples with variable thicknesses of collagen layer. Moreover, these observations imply that one can dramatically simplify the imaging and post-processing steps to reveal ablated tissue. This is shown in Fig. 10, which displays the outcomes of the spectral unmixing of Auf-HSI datasets containing 32 spectral bands vs. a simplified procedure that uses only two spectral bands. Specifically, the grayscale tiff image corresponding to 550 nm was divided by the tiff image corresponding to 490 nm. Since 490 nm is the wavelength where the intensity of most pixels is the highest, this procedure corresponds to spectra normalization and reveals the 550 nm elevation in the DBNS traces across all pixels of the image. The ablation lesions can then be displayed using either a grayscale, pseudocolor, or 3D mesh. To reduce the noise, one can also sum up images from adjacent spectral bands in the 520–580 range, which corresponds to the widening of the optical filter during the acquisition.

## Discussion

We have previously shown that Auf-HSI can be used to reveal RF ablation lesions in heart tissue from rats, pigs, and cows and that it works in blood-perfused preparations^8,9,11^. We have also shown that this approach works in the highly collagenous left atrium of aged humans^10^ and that it can reveal multiple targets, including myocardial scar, blood vessels, or adipose tissue accumulation^11^. Nonetheless, from several years of working with hundreds of samples, we have also noticed that spectral changes caused by RF ablation can vary significantly between individual animals and/or lesion sites, including cases when Auf-HSI was not able to reveal the ablated tissue. Thus, it was important for us to better understand the key factors that cause such variability. By doing so, we hoped to move closer to our ultimate goal of developing Auf-HSI into a reliable diagnostic tool that can be incorporated into the design of future ablation and/or imaging catheters.

The cartoon in Fig. 4a summarizes what we believe are the main factors behind spectral changes caused by RF ablation of cardiac tissue. These include the autofluorescence of endocardial collagen layer and the fraction of the illuminating light reflected back by the underlying ablated muscle layer, the drop of NADH fluorescence in live muscle, and the formation of yellow pigments by the heated muscle. The Fig. 4b graph shows an averaged DBNS trace from RF-ablated porcine LA, with the colored arrows pointing to spectral ranges where these individual factors are at play. The formation of pigments and the red shift due to muscle scattering both act to elevate the DBNS right shoulder. The loss of NADH and the increased scattering of collagen layer autofluorescence by ablated muscle have the opposite effects on the DBNS left shoulder, making this range more prone to sample variability, which can be clearly seen in the experimental traces shown in Fig. 3.

The degree of ablation-induced NADH loss in experimental settings depends not only on the amount of delivered RF energy but also on the way the LA tissue is handled and stored before ablation. Storage conditions have a relatively small impact on ablation-induced changes in the muscle reflectance or autofluorescence spectrum of the endocardial layer. On the other hand, muscle NADH levels first briefly increase due to the tissue becoming ischemic, followed by temperature- and time-dependent decline (Fig. 5). These changes can impact the pre-ablation levels of muscle NADH and lead to an unanticipated drop in NADH signal intensity. This has important practical implications. In most ablation studies, including ours, freshly excised pig or cow hearts (or donated human specimens) are put in ice-cold saline for transportation from an abattoir or a surgery facility to the lab. However, these hearts are quite big and cooling them takes time. As a result, the atria might stay warm for several hours, even when placed in an ice bucket, due to them being positioned next to a bulky warm ventricle. Therefore, to preserve LA fluorophores, it is recommended to detach the atria from the ventricle immediately after excision to maximize their exposure to an ice-cold solution during transportation to the lab.

It is important to clarify the difference between the decrease in NADH levels in metabolically active cells vs cells that are irreversible damaged by ablation. The marked drop in NADH autofluorescence caused by RF ablation is due to irreversible loss of cell viability which can be readily confirmed by post-ablation staining using TTC. Acute cell necrosis occurs because heat from application of RF energy raises tissue temperature to above 60 °C—a critical point when proteins start to coagulate, and muscle cells die^33–36^. Therefore, ablation induced NADH loss represents a different scenario from reversible drop in NADH levels when tissue metabolic rate increases leading to consumption of reducing equivalents^25,37^. Similarly, the observed formation and release of yellow pigments after ablation is not an outcome of metabolic changes since the cells are already ‘dead’. Multiple studies from the field of food science have shown formation of yellow pigments during meat cooking^31,32^. RF-ablation essentially ‘cooks’ cardiac muscle leading to formation of the heat-induced pigments. Detailed analysis of what is included in this complex mix lies outside the scope of this particular study.

Notably, experiments shown in Figs. 5 and 6 were not designed to mimic in vivo clinical scenarios. Instead, they helped us to better understand the observed variability of experimental traces from our bench experiments. They are still relevant for clinical implementation of spectral imaging since mixed or negative results obtained due to improperly stored tissue can halt the development of catheters based on this technology.

One of the most interesting findings of this paper is the role of diffuse reflectance of an underlying muscle layer in changing the surface autofluorescence profile of the ablated LA. In essence, UV illumination causes the endocardial collagen layer to act as a ‘lamp’ that shines visible light onto the underlying muscle. When the latter is ablated, more of these photons are scattered back, altering the overall tissue autofluorescence profile. Therefore, although the presence of collagenous endocardium masks the ablation-induced loss in muscle NADH, it also helps to reveal the ablation-induced changes in muscle diffuse reflectance. With increasing thickness of endocardium, the amplitude of this beneficial effect first goes up and then declines, as thicker collagen layers start blocking ablation-induced changes in the muscle below.

In an interesting twist, changes in diffuse reflectance might also help Auf-HSI to estimate the depth of the ablation lesions. Indeed, our previous studies^10^ revealed a strong correlation between the depth of the lesion (up to 6 mm) and the degree of spectral changes on the atrial surface (p < 0.001, R = 0.92). We believe that the observed correlation is a result of two, simultaneously occurring physical events. As the amount of RF energy increases, the lesion grows deeper. At the same time, the very surface of the heated muscle undergoes physical changes that causes further increase in scattering coefficient. The result is a predictive correlation: the more a tissue is thermally damaged from the surface, the deeper is the lesion. It is then possible to speculate that Auf-HSI can provide real-time information about the lesion’s depth. Moreover, considering that the average thickness of the human atrial wall is about 3 mm^9^, it might be also able to predict lesion transmurality. Additional experiments are required to confirm these hypotheses.

Our analysis considered only what we thought to be the major variables. Several additional factors may also be at play. One of them is the presence of cells, including smooth muscle cells, myofibroblasts, and endothelial cells, within the endocardium (the term ‘collagen layer” is an obvious simplification of a more complex structure)^38,39^. Applied RF energy destroys these cells, leading to the loss of their intracellular NADH. Relatively small amounts of these cells and a large amplitude of autofluorescence coming from collagen fibers mask this effect. Therefore, we did not observe any consistent changes in the profiles of endocardial layer taken from the top of the lesions. Yet it is possible that in vivo, a larger contribution from endocardial cells will be seen. Notably, one should not confuse ablation-induced NADH loss from viable cells present within the endocardium with a transient decrease in collagen autofluorescence upon heating^22^. In contrast to irreversible NADH decline in injured cells, collagen autofluorescence bounces back when the tissue cools to its original temperature. Although the latter phenomenon can be potentially useful for monitoring lesion formation *while* ablating^17,40^, our imaging studies are focused on permanent tissue damage, i.e. *after* the lesion has been formed and the tissue has returned to its pre-ablation temperature.

Another possible factor to consider is ablation-induced evaporation of water from the collagen layer, which occurs when ablations are performed on the excised specimen under open-air conditions. The main effect of this loss of moisture is an increase in the amplitude of both emitted and reflected light, which can make the lesion sites appear brighter^41^. Despite increased visibility to the human eye, such a drying effect does not improve lesion recognition based on spectral analysis. This is because HSI processing algorithms classify pixels based on differences in their *normalized* spectral profiles and not the amplitude of the spectrum. RF-induced moisture loss, however, can also lead to an increase in diffuse scattering within the endocardial layer, shifting spectral profiles to the right, similar to the effect caused by the reflective tape shown in Fig. 7. When RF ablations are performed in vivo, the impact of such drying-out effect is expected to be negligible, as water evaporation is minimized in the presence of surrounding blood and irrigating saline.

The ultimate goal of our studies is to determine how one might use Auf-HSI clinically. As such, concerns regarding levels of ultraviolet irradiation and its potential risks to the healthy tissue around the lesions has to be considered. In this regard, it is important to mention that the range of wavelengths best suitable for eliciting endogenous myocardial fluorescence is between 340 and 380 nm which falls within the UVA range of 315–400 nm. Compared to UVB or UVC, the UVA exposure causes the least damage due to its longer frequency which translates to lower photon energy. Secondly, our recent quantitative analysis^16^ has shown that the levels of UV needed for percutaneous Auf-HSI imaging are well below the established thresholds that can damage the skin. Lastly, the layer of endocardial collagen essentially shields the underlying muscle cells from direct UV exposure further minimizing any potential damage.

The important practical implication of our studies is the dramatically reduced amount of spectral information needed to reveal the ablated tissue. In the past, we have attempted to approach this issue mathematically^12,42^. In those studies, we used combinatorial tools to screen all possible wavelength combinations from the Auf-HSI datasets for a minimal number of wavelengths that can still provide good-quality outcomes. Notably, these numerical studies pointed to similar spectral ranges, yet the mechanisms behind these findings remained unclear until the experiments detailed in this paper were performed. Moreover, the presented new findings point to specific wavelength ranges from which photons can be lumped together. This information can be useful for the implementation of this technology into the design of percutaneous catheters to be used clinically. The limited size of the catheter lumen dramatically decreases the amount of light (both delivered and collected), while imaging the inner surfaces of a beating heart requires fast acquisition to minimize contraction artifacts. Both constraints severely limit the number of photons that get back to the detector. Thus, the ability to lump together multiple spectral bands by using custom-designed bandpass filters can increase the intensity of the signal.

In summary, the presented experimental data help explain the main variables behind the spectral changes caused by RF ablation of cardiac muscle covered by endocardial collagen layer of variable thickness. Our findings suggest a way to optimize the acquisition and post-processing steps by using a lesser number of spectral bands. The conclusions from our experiments can be used for diagnostic imaging of the heart and other multilayered tissues such as the skin, endovascular or epithelial surfaces. Ultimately, we aim to incorporate this technology into the design of percutaneous ablation catheters, with the hope of improving the outcomes of surgical procedures to treat atrial fibrillation and other cardiac arrhythmias.

## Data Availability

All data generated or analyzed during this study are included in this published article.

## Abbreviations

Auf-HSI: Autofluorescence hyperspectral imaging
DBNS: Difference between normalized spectra
LA: Left atrium
RF: Radiofrequency
UV: Ultraviolet
LCTF: Liquid crystal tunable filter

## References

[1] Wazni OM (2005). Radiofrequency ablation vs antiarrhythmic drugs as first-line treatment of symptomatic atrial fibrillation: A randomized trial. J. Am. Med. Assoc..

[2] Haïssaguerre M (2005). Catheter ablation of long-lasting persistent atrial fibrillation: Critical structures for termination. J. Cardiovasc. Electrophysiol..

[3] Lee G, Sanders P, Kalman JM (2012). Catheter ablation of atrial arrhythmias: State of the art. Lancet.

[4] Ganesan AN (2013). Long-term outcomes of catheter ablation of atrial fibrillation: A systematic review and meta-analysis. J. Am. Heart Assoc..

[5] Cappato R (2010). Updated worldwide survey on the methods, efficacy, and safety of catheter ablation for human atrial fibrillation. Circ. Arrhythm. Electrophysiol..

[6] Sánchez-Quintana D, Doblado-Calatrava M, Cabrera JA, Macías Y, Saremi F (2015). Anatomical basis for the cardiac interventional electrophysiologist. BioMed Res. Int..

[7] Haïssaguerre M (1998). Spontaneous initiation of atrial fibrillation by ectopic beats originating in the pulmonary veins. N. Engl. J. Med..

[8] Gil DA (2017). Autofluorescence hyperspectral imaging of radiofrequency ablation lesions in porcine cardiac tissue. J. Biophotonics.

[9] Muselimyan N, Jishi MA, Asfour H, Swift L, Sarvazyan NA (2017). Anatomical and optical properties of atrial tissue: Search for a suitable animal model. Cardiovasc. Eng. Technol..

[10] Muselimyan N (2016). Seeing the invisible: Revealing atrial ablation lesions using hyperspectral imaging approach. PLoS One.

[11] Swift LM (2018). Hyperspectral imaging for label-free in vivo identification of myocardial scars and sites of radiofrequency ablation lesions. Hear. Rhythm.

[12] Guan S, Asfour H, Sarvazyan N, Loew M (2018). Application of unsupervised learning to hyperspectral imaging of cardiac ablation lesions. J. Med. Imaging.

[13] Mercader M (2012). Use of endogenous NADH fluorescence for real-time in situ visualization of epicardial radiofrequency ablation lesions and gaps. Am. J. Physiol. Heart Circ. Physiol..

[14] Swift LM (2014). Visualization of epicardial cryoablation lesions using endogenous tissue fluorescence. Circ. Arrhythm. Electrophysiol..

[15] Mercader M (2013). Real-time NADH fluorescence imaging catheter (LuxCath): A novel imaging system for evaluation of radiofrequency ablation lesions and gaps. Hear. Rhythm A.

[16] Armstrong K, Larson C, Asfour H, Ransbury T, Sarvazyan N (2020). A percutaneous catheter for in vivo hyperspectral imaging of cardiac tissue: Challenges, solutions and future directions. Cardiovasc. Eng. Technol..

[17] Koruth J (2015). Direct assessment of catheter-tissue contact and RF lesion formation: A novel approach using endogenous NADH fluorescence. Hear. Rhythm.

[18] Kitzman DW, Edwards WD (1990). Age-related changes in the anatomy of the normal human heart. J. Gerontol..

[19] Schwartzman D, Schoedel K, Stolz DB, Di Martino E (2013). Morphological and mechanical examination of the atrial ‘intima’. Europace.

[20] Chance B (2004). Mitochondrial NADH redox state, monitoring discovery and deployment in tissue. Methods Enzymol..

[21] Wu Y, Xi P, Qu JY, Cheung T-H, Yu M-Y (2004). Depth-resolved fluorescence spectroscopy reveals layered structure of tissue. Opt. Express.

[22] Walsh AJ, Masters DB, Jansen ED, Welch AJ, Mahadevan-Jansen A (2012). The effect of temperature on the autofluorescence of scattering and non-scattering tissue. Lasers Surg. Med..

[23] Bowen WJ (1949). The absorption spectra and extinction coefficients of myoglobin. J. Biol. Chem..

[24] Croce AC, Bottiroli G (2014). Autofluorescence spectroscopy and imaging: A tool for biomedical research and diagnosis. Eur. J. Histochem..

[25] Asfour H (2012). NADH fluorescence imaging of isolated biventricular working rabbit hearts. J. Vis. Exp..

[26] Swift LM (2008). Controlled regional hypoperfusion in Langendorff heart preparations. Physiol. Meas..

[27] Kay MW, Swift LM, Martell B, Arutunyan A, Sarvazyan NA (2008). Locations of ectopic beats coincide with spatial gradients of NADH in a regional model of low-flow reperfusion. Am. J. Physiol. Hear. Circ. Physiol..

[28] Aliverti A, Curti B, Vanoni MA (1999). Identifying and quantitating FAD and FMN in simple and in iron-sulfur-containing flavoproteins. Methods Mol. Biol..

[29] Pollegioni L (2003). Contribution of the dimeric state to the thermal stability of the flavoprotein D-amino acid oxidase. Protein Sci..

[30] Jamieson JD (1964). Specific granules in atrial muscle cells. J. Cell Biol..

[31] Gatellier P, Santé-Lhoutellier V, Portanguen S, Kondjoyan A (2009). Use of meat fluorescence emission as a marker of oxidation promoted by cooking. Meat Sci..

[32] Lund MN, Heinonen M, Baron CP, Estévez M (2011). Protein oxidation in muscle foods: A review. Mol. Nutr. Food Res..

[33] Thomsen S, Jacques S, Flock S (1990). Microscopic correlates of macroscopic optical property changes during thermal coagulation of myocardium. Laser-Tissue Interact..

[34] Rempp H (2009). Prediction of cell necrosis with sequential temperature mapping after radiofrequency ablation. J. Magn. Reson. Imaging.

[35] Yosshimura H, Viator JA, Jacques SL (2005). Relationship between damaged fraction and reflected spectra of denaturing tissues. Lasers Surg. Med..

[36] Nagarajan VK, Yu B (2016). Monitoring of tissue optical properties during thermal coagulation of ex vivo tissues. Lasers Surg. Med..

[37] Moreno A, Kuzmiak-Glancy S, Jaimes R, Kay MW (2017). Enzyme-dependent fluorescence recovery of NADH after photobleaching to assess dehydrogenase activity of isolated perfused hearts. Sci. Rep..

[38] Park JH (2013). The clinical significance of the atrial subendocardial smooth muscle layer and cardiac myofibroblasts in human atrial tissue with valvular atrial fibrillation. Cardiovasc. Pathol..

[39] Lannigan RA, Zaki SA (1966). Ultrastructure of the normal atrial endocardium. Br. Heart J..

[40] Armstrong K (2015). Comparison of optical tissue interrogation vs impedance measurement for real-time monitoring of catheter-tissue contact and RF lesion progression. Hear. Rhythm.

[41] Zhu Q, Shen Y, Zhang A, Xu LX (2013). Numerical study of the influence of water evaporation on radiofrequency ablation. Biomed. Eng. Online.

[42] Asfour H (2018). Optimization of wavelength selection for multispectral image acquisition: A case study of atrial ablation lesions. Biomed. Opt. Express.

